# Evaluation of implant abutment screw head deformation in hexagonal and star designs after successive tightening and loosening

**DOI:** 10.34172/japid.2023.001

**Published:** 2022-08-21

**Authors:** Tahereh Ghaffari, Amin Nourizadeh, Elnaz Shafiei, Shima Ghasemi, Mohammadhasan Mahmoudzadeh, Kosar Ataei

**Affiliations:** ^1^Department of Prosthodontics, Faculty of Dentistry, Tabriz University of Medical Sciences, Tabriz, Iran; ^2^Department of Prosthodontics, Faculty of Dentistry, Tabriz Medical Sciences, Islamic Azad University, Tabriz, Iran; ^3^Private Practice, Tabriz, Iran; ^4^Dental Student, Faculty of Dentistry, Tabriz University of Medical Sciences, Tabriz, Iran

**Keywords:** Abutment, Surface area change, Implant, Screw

## Abstract

**Background.:**

Tightening and loosening of the prosthetic components of the implant are carried out with various screw designs. This study compared the rate of deformation of the abutment screw in two hexagonal and star screw head designs after consecutive tightening and loosening.

**Methods.:**

In this study, two fixtures were mounted vertically in die stone blocks using a surveyor. Then the corresponding abutment (with a diameter of 4.5 mm and a gingival height of 2 mm; Dio-SM and Dio-UF system) was mounted on each fixture. In each fixture, six abutment screws from each manufacturer were used (12 abutment screws in total). The abutment screw head of the Dio-UF system is hexagonal, and the abutment screw head of the Dio-SM system is star-shaped. The samples were examined under a stereomicroscope (Nikon C-DS) at a magnification of×50. The image of each abutment screw head (6 abutment screws in each group) was prepared before the procedural steps and after 5, 10, and 15 times of tightening and loosening with 25-Ncm torque using a stereomicroscope. Then the differences in the surface areas of consecutive specimens (0, 5, 10, and 15 consecutive tightening and loosening rounds) between hexagonal and star-shaped abutment screws were calculated. The study results were reported via descriptive statistical methods (mean±standard deviation). Statistical analysis was performed using SPSS 24, and the significance level was defined at *P*<0.05.

**Results.:**

Increasing the number of tightening and loosening rounds increased the screw head surface area in both hexagonal and star shapes. At all stages, the changes in the star-shaped screw head were greater than in the hexagonal screw. These changes were statistically significant at all stages (*P*<0.05). In addition, there was a statistically significant difference between the area values and the number of tightening and loosening rounds separately in both screw types (*P*<0.001). Also, the surface areas of the head of both screws in all tightening and loosening rounds were significantly different (*P*<0.001).

**Conclusion.:**

Increasing the number of tightening and loosening rounds increased the screw head surface area in both hexagonal and star-shaped geometrical forms. Also, the extent of area changes at all stages in star-shaped screw heads was greater than in hexagonal screws.

## Introduction

 It is very important to replace missing or lost teeth. Failure to do so can cause esthetic and functional problems, speech disorders, and temporomandibular joint problems. Dental implants replace missing or dysfunctional teeth; they are surgically placed in the bone.^1^ The reconstruction of dental structures and implant-based prostheses has been a great success.^2^ For example, in a 5-year study, Jung et al^3^ reported a 94.5% success rate for single implant-based prostheses.

 The connection of the abutment to the fixture significantly affects the success of the implant prosthesis. In the most common method of mechanical connections, screw abutments are used to connect the abutment to the implant.^4^ In general, the reliability and stability of the implant and abutment connection is a necessary prerequisite for the success of dental implants.^5^

 Implant-abutment junction design can affect screw loosening, soft and hard tissue preservation, and leakage into the implant site.^6^ The coping screw is usually the weakest joint in the prosthetic chain. Any malalignment in the occlusion, cast adaptation, or force can lead to loosening or fracture of the screw during function. These problems can protect the body of the implant against more serious complications. When these problems occur in a splinted restoration, other implant abutments are exposed to the risk of overloading and more problems than the implant in question because a cantilever and force intensifier is created.^7-9^

 Screw fracture and loosening are among the most critical challenges of implant prosthetics that have been considered in research today.^10^ Fracture and loosening of the screw are related to the components, metal fatigue, intermittent movements during function, non-axial pressure, bone elasticity, and the amount of torque and preload.^11,12^

 Preload depends on various factors, such as the design of the screws and threads, the surface roughness, the materials used, and the applied torque. Adequate torque is vital for preventing screw loosening.^13^ The excessive increase in torque can reduce the proportional limit in the screw and cause loosening and permanent deformation of the screw; it also decreases torque, which results in loosening and fatigue of the screw.^14,15^

 In case of successive unscrewing of the screw, surface changes in the screw head‒screwdriver and abutment‒screw interfaces will be inevitable. Changes in the screw head due to fracture are a potential problem.^16^

 Guzaitis et al^17^ proposed that the clinical expectation of a screw cap could be met by a maximum of 10 screw loosening rounds, after which the screw should be discarded, and a new screw should be used. Various screw designs have been proposed by implant manufacturers to solve this problem, including hex-slotted, star, and square-slotted hexagons.

 Kim et al^16^ reported increased scratches during the placement of the square-shaped wrench over the hex-slotted shape. However, square-head screws performed better concerning fracture resistance and had a lower fracture rate than hexagonal screws.

 De Paiva et al^2^ reported no significant difference in the elastic deformation in the square and hexagonal designs after 10 times of tightening and loosening with 32-Ncm torque.

 After loosening the screw, it is necessary to re-tighten or replace the abutment screw with another screw, but in some cases, more measures are required.^14^ Clinically, the possibility of tightening and loosening the abutment screw in implant-based prostheses is significant.^18^

 Considering the high prevalence of screw loosening as a result of successive tightening and loosening of the abutment screw, and given the lack of sufficient data on the fracture of the screw head and the contradictory results in the existing studies, we aimed to compare the rate of deformation of the abutment screw in two hexagonal and star screw head designs in consecutive tightening and loosening.

## Methods

 In this study, performed in the School of Dentistry, Tabriz University of Medical Sciences, two fixtures with a diameter of 4.1 mm and a length of 11.5 mm from two different types of an implant system, Dio-SM and Dio-UF (Dio, Seoul, Korea), were used.

 Using a surveyor, the fixtures were then fixed vertically in die stone blocks (GC FUJIROCK^®^ EP Corporation, Tokyo, Japan) made in a standardized metal mold measuring 50 × 20 × 20 mm. The gypsum blocks in which the fixtures were mounted were fixed on a table holder. Then on each fixture, the corresponding abutment (with a diameter of 4.5 mm and a gingival height of 2 mm from the Dio-SM and Dio-UF systems) was mounted. Also, a 25-Ncm torque was applied with the abutment wrenches of Dio-SM and Dio-UF companies according to the manufacturers’ recommendations.

 In each fixture, six abutment screws from each manufacturer were used (12 abutment screws in total). The abutment screw head of the Dio-UF system is hexagonal, and the abutment screw head of the Dio-SM system is star-shaped. The samples were examined under a stereomicroscope (Nikon C-DS) at a magnification of × 50.

 The image of each abutment screw head (6 abutment screws in each group) was prepared before starting the procedural steps and after 5, 10, and 15 times of tightening and loosening with 25-Ncm torque using a stereomicroscope.

**Figure 1:**
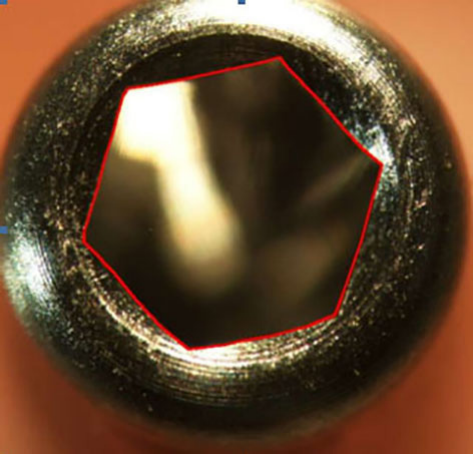
The lines drawn for the hexagonal screw in AutoCAD

**Figure 2:**
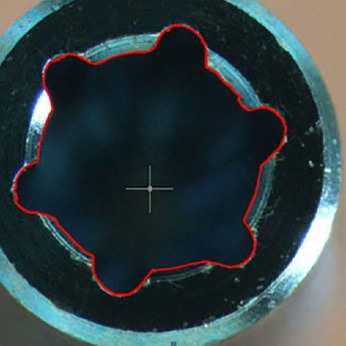
The lines drawn for the star screw in AutoCAD

**Figure 3:**
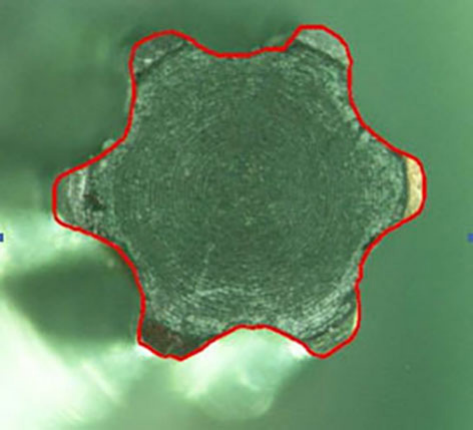
The shape of the screwdriver head corresponds to the star-shaped abutment screw head

**Figure 4:**
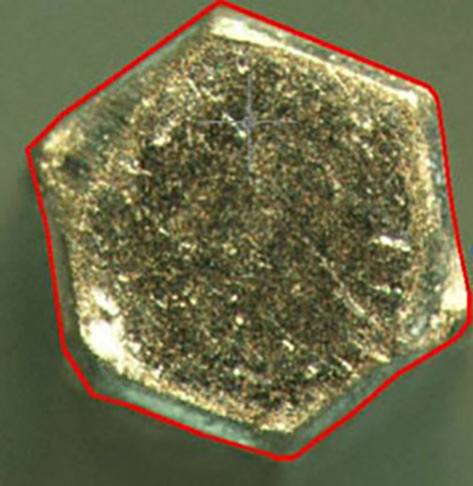
The shape of the screwdriver head corresponds to the hexagonal-shaped abutment screw head

 To record the difference in the amount of abutment head deformation in different designs, the outline shape of the abutments was drawn by AutoCAD software (Autodesk, Ink, SanRafael, California), and the surface areas of geometric shapes were calculated in mm^2^ (Figures 1-4). Then the differences in surface areas of consecutive specimens (0, 5, 10, and 15 consecutive tightening and loosening rounds) between hexagonal and star-shaped abutment screws were calculated.^16^

 The results were reported using descriptive statistical methods (mean ± standard deviation). First, the normality of data distribution was investigated to compare the difference in the area of the abutment head design in hexagons and stars. Then, repeated-measures ANOVA, Mauchly test, Bonferroni test, and t-test were used to analyze the differences between the groups. Finally, statistical analysis was performed using SPSS 24, and the significance level was defined at *P *< 0.05.

## Results

 An increase in the number of tightening and loosening rounds of the screws increased the surface area of the abutment screw head in both hexagonal and star geometric forms, increasing the wear in the hexagonal abutment screw from 0 to 15 rounds to 33.01 units and to 54.26 units in the star abutment screw. Also, in all the tightening and loosening rounds, the hexagon abutment screw surface area was less than that of the star-shaped abutment screw. The difference in the abutment screw surface areas between the two types of abutment screws in all the tightening and loosening rounds was statistically significant (*P* < 0.001).

 According to Table 1, the change in the surface area of the abutment screw in both hexagonal and star geometric forms from round 5 to round 10 exhibited the highest mean. The minimum changes in both forms were related to rounds 10 to 15.

**Table 1:** Surface area changes of the hexagonal and star-shaped abutment screw heads in three rounds of tightening and opening (mm^2^)

**Tightening & opening** **rounds of the screw head**	**Surface area changes (Mean±SD)**		***P*** ** value**
	**Star-shaped**	**Hexagonal**	
0-5	17.05 ± 0.57	7.74 ± 0.19	< 0.001
5-10	24.13 ± 0.32	18.56 ± 0.25	< 0.001
10-15	13.02 ± 0.63	6.69 ± 0.17	< 0.001

SD: standard deviation

 Repeated-measures ANOVA was used to compare the surface area of the hexagonal abutment screw design after 5, 10, and 15 rounds of tightening and loosening. For this purpose, this test was used separately in each type of abutment screw. According to the results, there was a statistically significant difference between surface area values in terms of the number of tightening and loosening rounds separately in each abutment screw design (*P* < 0.001). Also, due to the significance of the results of the Mauchly test in both groups, the test for the sphericity assumption was used for comparisons.

## Discussion

 In this study, two fixtures of the same diameter and length (4.1 mm in diameter and 11.5 mm in length) from different types of an implant system (Dio-SM and Dio-UF) were used. Abutments related to each system were attached to the fixtures with the same diameter and length (diameter of 4.5 mm and a gingival height of 2 mm). To determine the effect of the number of consecutive tightening and loosening rounds on the deformation of the implant abutment screw, six abutment screws specific to each system in the hexagonal (related to Dio-UF) and star (related to Dio-SM) designs were used on the respective abutments.

 The surface area changes in the star design in all the stages were greater than those in the hexagonal screw. The difference in the surface area change in all the phases in the star design was significantly greater than that in the hexagonal head.

 Since the contact surface of the star-shaped screw head is larger than the hexagonal screw, the contact surface area of the wrench in the star screw head design is higher, and its fracture is more severe.

 It seems that the fracture of the star screw can accelerate rapidly as a result of use. Studies on the abutment screw changes are very limited.

 De Paiva et al^2^ examined the resistance of square and hexagonal shapes in screw attachments to deformation and reported that the geometric shapes of screws did not affect their deformation. The difference in the results might be attributed to the methodology, the implant brands, and differences in the corresponding screw head designs.

 Kim et al^16^ found that the screw head design could affect the odds of fracture and deformation of the abutment head screw. Accordingly, the fracture in the head with a star design is more probable and more severe than the square head design, consistent with the present study.

 Kim et al^19^ examined changes in the screw abutment after repeated tightening and loosening rounds. There were several scratches near the screw head slot, possibly due to the repeated contact between the driver tip around the screw slot when the driver tip repeatedly tightened or loosened the screw. This was more noticeable in the square screw than in the hexagon. They also concluded that screws with a square design were better than hexagonal screws in the transmission and the effectiveness of screw tightening. According to this study, screws with a square slot design were more resistant to fracture or distortion than hexagonal screws.

## Conclusion

 According to the findings of the study in general:

1. By increasing the tightening and loosening rounds, the surface area of the abutment head increased in both hexagonal and star geometric forms. The results also showed a statistically significant difference between tightening and loosening rounds in both groups (*P* < 0.001).
2. The change in the surface area of the star design of the abutment screw in all the stages was more than the hexagonal screw of the abutment.

The highest increase in the surface area in both types of abutment screws was in 5‒10 rounds of tightening and loosening.

## Acknowledgments

 This study was supported by a grant from the Dental and Periodontal Research Center, Tabriz University of Medical Sciences.

## Availability of Data

 The datasets used and/or analyzed during the current study are available from the corresponding author on reasonable request.

## Competing Interests

 The authors declare that they have no competing interests related to authorship and/or publication of this work.

## Ethical Approval

 The study protocol was approved by the Ethics Committee on Medical Research, Tabriz University of Medical Sciences.

## Funding

 The authors received no financial support for the research, authorship, and/or publication of this article.
